# Biased computation of probability of target attainment for antimicrobial drugs

**DOI:** 10.1002/psp4.12929

**Published:** 2023-04-06

**Authors:** Pierre‐Louis Toutain, Peggy Gandia, Ludovic Pelligand, Aude A. Ferran, Peter Lees, Alain Bousquet‐Mélou, Didier Concordet

**Affiliations:** ^1^ INTHERES, Université de Toulouse, INRAE, ENVT Toulouse France; ^2^ The Royal Veterinary College University of London London UK

## Abstract

The medical literature is replete with articles in which there is confusion between “free concentration” and “unbound fraction” (*f*_u_), which is the ratio of free to total plasma concentration. The lack of clarity in distinguishing between these two terms has led to biased computations, erroneous interpretations, and misleading recommendations. The problems are highlighted in this paper, taking the example of calculation of Probability of Target Attainment (PTA). This metric is used to propose pharmacokinetic/pharmacodynamic (PK/PD) breakpoints required for the interpretation of Antimicrobial Susceptibility Testing. Based on Monte Carlo simulations of the PK/PD index, area under the unbound concentration time curve/minimum inhibitory concentration (*f*AUC/MIC), computation of PTA from total plasma concentrations scaled by *f*_u_ ineluctably leads to biased estimates. The bias is greater if the variability associated with *f*_u_ is added, instead of removing it during this scaling. The explanation for the bias is that total plasma drug concentrations are intrinsically more variable than the corresponding free concentrations. This is due to the variability of antimicrobial binding for total, but not for free plasma concentrations. In consequence, the greater variability always leads to underestimation of the PK/PD cutoff (i.e., the critical MIC that is guaranteed for a given percentile of the population). A further consequence is an increase in calculated dosage required to attain the targeted quantile. This erroneous approach, of using free antimicrobial drug fraction, is not limited to the derivation of PK/PD cutoff, but may also have consequences for antimicrobials drug safety in clinical patients.

**Study Highlights**

- WHAT IS THE CURRENT KNOWLEDGE ON THE TOPIC?

For antimicrobial drugs (antimicrobials), the active concentration is the free plasma concentration. It is routinely obtained from the total measured concentration using the unbound fraction (*f*_u_), as a scaling factor. Such transformation is used for the estimation of the Probability of Target Attainment (PTA) of an antimicrobial pharmacokinetic/pharmacodynamic index.

- WHAT QUESTION DID THIS STUDY ADDRESS?

To demonstrate, using Monte Carlo simulation, that the transformation of total plasma concentration into corresponding free concentration, leads unavoidably to biased estimates of PTA.

- WHAT DOES THIS STUDY ADD TO OUR KNOWLEDGE?

For a PTA of 90%, the computed area under the unbound concentration time curve/minimum inhibitory concentration distributions were always negatively biased. The bias is greater when the assumed variability of *f*_u_ is erroneously taken into account for scaling.

- HOW MIGHT THIS CHANGE DRUG DISCOVERY, DEVELOPMENT, AND/OR THERAPEUTICS?

When PTA is computed to support either dosage regimen determination or for Antimicrobial Susceptibility Testing (AST), the data are biased, leading to the risk of proposing a higher than necessary dose or enforcing AST clinical breakpoints which are too conservative.

## INTRODUCTION

For the vast majority of drugs, including antimicrobials, the active concentration, which determines pharmacological actions and clinical efficacy, is the unbound (or free) plasma concentration. However, for most antimicrobials, it is the total plasma concentration which is routinely measured and the free microbiologically active concentration is subsequently derived from this concentration using, as a scaling factor, the free/unbound fraction (*f*_u_). When drug binding to plasma proteins is linear, that is not concentration‐dependent, *f*_u_ is used to calculate the free plasma concentration (Equation 1):

(1) $$C_{\text{free}}=\mathrm{fu}\times C_{\mathrm{tot}}$$

where *Cfree* is the calculated free plasma concentration, *Ctot* the measured total plasma concentration and *fu*, the scalar with a value in the range of 0 to 1.

A classic example of such transformation is the estimation by Monte Carlo Simulations (MCSs) of the Probability of Target Attainment (PTA), for a given pharmacokinetic/pharmacodynamic (PK/PD) index, of a selected PD target.^1^, ^2^ These simulations are typically performed to determine PK/PD cutoffs (or PK/PD breakpoints) which are then used to set clinical breakpoints. The latter enable clinical microbiology laboratories to categorize microorganisms as clinically susceptible, intermediate, or resistant, using the standards of the European Committee on Antimicrobial Susceptibility Testing (EUCAST),^3^ the Clinical and Laboratory Standards Institute (CLSI)^4^, ^5^ and other organizations, such as USCAST.

For antimicrobials, the two most commonly used PK/PD indices are *f*T> minimum inhibitory concentration (MIC), the time over which free plasma concentration exceeds the MIC, usually expressed as a percentage of a 24 h dosing interval (%*f*T>MIC), and *f*AUC/MIC, the ratio of the area under the curve of free plasma concentration over 24 h at steady‐state to MIC. For both indices, the italicized *f* indicates that calculations are made with the free plasma concentration.

MCSs are conducted using previously obtained PK parameters to generate predictions of outcomes for several dosing regimens.^6^ PK parameters are obtained from population PK (PopPK) investigations, conducted in healthy volunteers or preferably in patients and followed by PopPK modeling using a nonlinear mixed effects model. To the best of our knowledge, for almost all PopPK investigations, the quoted *f*_u_ values are not determined at the time, and individual free plasma concentrations are usually not directly obtained from measured individual total plasma concentrations. Rather, when *f*_u_ is reported, it is obtained from satellite investigations, in which *f*_u_ is generally derived from pooled plasma samples, thus giving an “average” *f*_u_. Less frequently, individual plasma samples are used to provide a distribution of individual *f*_u_ values.^7^

For MCS modeling, an average *f*_u_ value is selected, a priori, from the scientific literature, the product package labeling^5^ or as stated in product monographs.^8^ Some authors have also included, in their simulations, the variability associated with *f*_u_. For example, Zelenitsky et al.^8^ transformed total concentration data into free concentrations using Gaussian distributions of *f*_u_ of 0.93+/−0.02 for meropenem, 0.70+/−0.02 for piperacillin/tazobactam, 0.85+/−0.02 for cefepime, and 0.85+/−0.02 for ceftobiprole, as presented in the product monographs. For PK/PD analyses for 13 antimicrobials from six antimicrobial classes, with actions against *Pseudomonas aeruginosa* and *Acinetobacter baumannii*, plasma protein binding was assumed to vary according to an a priori uniform distribution (±10%).^5^ A uniform distribution of (+/−10%) was also used for the comparison of PK/PD breakpoints with EUCAST and CLSI clinical breakpoints of Gram‐positive bacteria.^9^

When *f*AUC/MIC is the selected PK/PD index, the most frequent approach is to scale the PK/PD index value with total plasma concentration (i.e., *total*(*AUC/MIC*), by an “average” *f*_u_ to obtain the corresponding and final PK/PD index value). This is expressed as free plasma concentration (i.e., *f*AUC/MIC). For *f*T>MIC, total plasma concentration data are transformed into corresponding free plasma concentrations before computation of the *f*T>MIC.

The aim of this paper is to demonstrate that transforming total into free plasma concentration, using an average value of *f*_u_ to derive PTA, leads inevitably to biased estimates for 90% PTA. Moreover, this bias increases if the variability of *f*_u_ is included in the model.

## RATIONALE

Both low and high quantile values of *f*AUC/MIC and %*f*T>MIC distributions, as currently computed with *f*_u_, are almost invariably biased. This occurs because total plasma concentrations are intrinsically more variable than the corresponding free plasma concentrations, in consequence of the variability in antimicrobial binding to plasma proteins. When a drug is administered intravenously, the steady‐state free plasma concentration is controlled only by free plasma clearance. The free steady‐state plasma concentration, ${\text{Cfree}}_{\mathrm{ss}}$, following continuous intravenous infusion, is given by Equation 2:

(2) $${\text{Cfree}}_{\mathrm{ss}}=\frac{\mathrm{INF}\left(\text{rate}\right)}{{\mathrm{CL}}_{\text{free}}}$$

with INF(rate), the continuous infusion rate (e.g., in μg/kg/h) and *CLfree*, the free plasma clearance (ml/kg/h) giving, at equilibrium, a free steady‐state plasma concentration ${\text{Cfree}}_{\mathrm{ss}}$ in μg/ml.^10^ Assuming a constant infusion rate, Equation 2 indicates that the between subject variability (BSV) of *Cfree*_*ss*_ is due to a single source of variability, namely the BSV of free plasma clearance. It is also important to note that, for low extraction ratio drugs (including almost all antimicrobials), free plasma clearance is independent of *f*_u_. In contrast, total plasma concentration (*Ctot*) is controlled by both free plasma concentration and the extent of drug binding to plasma proteins^11^ (Equation 3):

(3) $$\text{Ctot}=\text{Cfree}+\frac{\text{Bmax}\times \text{Cfree}}{\mathrm{Kd}+\text{Cfree}}$$

with *Bmax*, the maximal binding capacity (which has the same units as *Cfree* or *Ctot*) reflecting the molar concentration of plasma binding proteins and *Kd* (the same units as *Bmax*, *Cfree* and *Ctot*), the equilibrium dissociation constant, reflecting drug affinity for plasma binding proteins.

When *Kd*> > *Cfree*, Equation 3 simplifies to Equation 4:

(4) $$\text{Ctot}=\text{Cfree}\times \left(1+\frac{\text{Bmax}}{\mathrm{Kd}}\right)$$

In Equations 3 and 4, *Ctot* is the dependent variable, *Cfree* is the independent variable, and *Bmax* and *Kd* are the two binding parameters. Therefore, the variability of *Ctot* is the combination of the variabilities of *Cfree* (attributable to that of *CLfree*) and those of *Bmax* and *Kd*. Indeed, *Bmax* and *Kd* display BSV, reflecting for *Bmax* the variability of molar concentration of plasma binding proteins.

When simulating and interpreting variations of *Cfree* and *Ctot*, it is Equations (2), (3), (4) that must be considered and not Equation 1. Equation 1 is simply an operational relationship among *f*_u_, *Cfree*, and *Ctot*, and it should not be used for any physiological interpretation or simulation.^11^ For example, it is frequently reported, using Equation 1, that an increase of *f*_u_ causes an increase *Cfree*. This is wholly incorrect in in vivo situations, because only Equation 5 defines the mechanistic dependency of *f*_u_:

(5) $$\mathrm{fu}=\frac{\text{Cfree}}{\text{Ctot}}=\frac{\mathrm{Kd}}{\text{Bmax}+\mathrm{Kd}}$$

which is obtained by incorporating the expression of Ctot when *Kd*> > *Cfree* (Equation 4), and Equation 6 allows physiological interpretation of a change in *f*_u_:

(6) $$\text{Ctot}=\frac{\text{Cfree}}{\mathrm{fu}}$$

Any alteration of *f*_u_ (due to *Bmax* and/or *Kd*, as in Equation 5) can only impact on total, and not free, plasma concentration, the latter being independent of plasma protein binding (Equation 2). Any increase or decrease in *f*_u_ is due to a decrease or an increase of *Ctot*, respectively (Equation 6), not an increase or a decrease of *Cfree*, as misleadingly suggested by Equation 1. These considerations have been established in previous publications.^10^, ^12^, ^13^

The second widely used PK/PD index, especially for beta‐lactam antimicrobials, is %*f*T>MIC. For this index, it was noted during our simulations, incorporating sensitivity analysis, that the biases resulted from a complicated interplay among the numerical values of the parameters, their BSV, the MIC to be achieved, the dose, dosing interval, and targeted PTA. The purpose of this paper is to draw attention to the principle that transforming distributions of total plasma concentration into distributions of corresponding free plasma concentration (or PK parameters derived therefrom) is intrinsically biased. To avoid extensive detail, while retaining the general principles of the argument, the paper is restricted to results obtained for the PK/PD *f*AUC/MIC index.

## METHODS

MCSs were carried out using Oracle Crystal Ball (CB); release 11.1.2.4900. An R‐script has also been developed to allow the CB results to be reproduced with open access software (see supportive information).

A reference distribution of *f*AUC/MIC (*n* = 5000) was simulated using Equation 7 and solved with a dose of 10 mg/kg and a lognormal distribution of clearance, with a mean of 7 L/kg/day and a standard deviation of 0.7 L/kg/day, corresponding to a coefficient of variation (CV) = 10% (i.e., Ln(CLFree) ~ N(1.9409,0.09975^2^)).

(7) $$\frac{\text{fAUC}}{\mathrm{MIC}}=\frac{\text{Dose}}{\text{CLFree}\times \mathrm{MIC}}$$

MIC was fixed to 1 mg/L without loss of generality. The 10% quantile (Q10%) of *f*AUC/MIC distribution (here 1.26 days or equivalently 30.2 h, according to the usual means of expressing the target PK/PD index in h) is the reference Q10% true value corresponding to the PTA90%. This reference distribution was transformed into the corresponding distribution of the total AUC/MIC, denoted *total*(*AUC/MIC*), (i.e., into the distribution which is usually observed; Equation 8):

(8) $$\frac{\text{totalAUC}}{\mathrm{MIC}}=\frac{\text{fAUC}}{\mathrm{MIC}}\times \frac{1}{\mathrm{fu}}$$

Two scenarios were simulated to compute reference *total*(*AUC/MIC*) distributions: one scenario for an average *f*_u_ of 0.5 (moderate binding) with a CV of 15% (i.e., with Ln(*fu*) ~ N(−0.704272, 0.149166^2^) and a second scenario (high binding) with a uniform distribution of *f*_u_ ranging from 0.05 to 0.15. Finally, the corresponding back‐computed *f*(*back*)AUC/MIC were derived from reference *total*(*AUC/MIC*) distributions using Equation 9, as would be undertaken for a real‐life scenario. We computed *f*(*back*)AUC/MIC both for a given *f*_u_ (scalar, either 0.5 or 0.1) or an *f*_u_ distribution (either 0.40 to 0.60 rather than a scalar of 0.5 or 0.05–0.15 rather than a scalar of 0.1). This enabled estimation of Q10%, as routinely conducted when free plasma concentration is not available.

(9) $$\begin{array}{c} f\left(\text{back}\right)\mathrm{AUC}/\mathrm{MIC}=\left[\;\mathrm{fu}\left(\text{scalar}\right)\text{or}\;\mathrm{fu}\left(\text{distribution}\right)\right]\\ \;\times \text{total}\left(\mathrm{AUC}/\mathrm{MIC}\right)\left(\text{distribution}\right) \end{array}$$

Units selected for AUC (mg*day/L) enabled Q10% results to be reported in units of days, even when the units implicitly used but generally not reported to express this PK/PD index are hours.^14^ The advantage of expressing AUC/MIC in days (1.26 day rather than 30.2 h) is to report the numerical value of the average plasma concentration over 24 h actually achieved in steady‐state conditions and the reference Q10% corresponds to an average free plasma concentration of 1.26 mg/L over 24 h (see Toutain et al.^15^ for explanation).

Bias of Q10% obtained by scaling *total*(*AUC/MIC*) with *f*_u_ of 0.5 or 0.1 or *f*_u_ distributions with the reference free Q10% obtained with *f*(*back*)AUC/MIC was computed with Equation 10:

(10) $$\begin{array}{c} \text{Bias}\%=100\times \frac{\text{Scaled}\_Q10\text{AUCtotal}/\mathrm{MIC}-\mathrm{Ref}\_Q10\text{AUCfree}/\mathrm{MIC}}{\mathrm{Ref}\_Q10\text{AUCfree}/\mathrm{MIC}} \end{array}$$

Beta‐lactam antimicrobials are administered by prolonged constant intravenous infusion as a means of improving efficacy.^16^ For infusion, Equation 2 was used to simulate unbound steady‐state concentration (*f*Css). It should be noted that Equation 2 is algebraically identical to Equation 7, which was used to generate *f*AUC, the dose (mass units) of Equation 7 being replaced by an infusion rate (mass unit per time) in Equation 2 and the dependent variable being now a Css rather than an *f*AUC distribution. No other simulations than those performed for AUC/MIC are necessary to highlight bias associated with continuous infusions (see Discussion).

**FIGURE 1:**
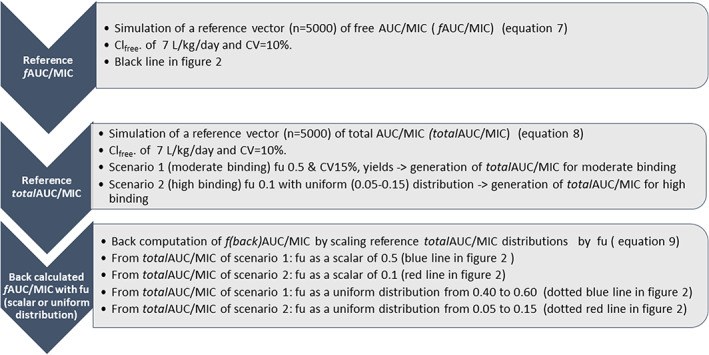
Simulation of reference vectors of free and total reference AUC/MIC and back calculated distributions. AUC, area under the concentration time curve; Cl, clearance; CV, coefficient of variation; *f*AUC, area under the unbound concentration time curve/minimum inhibitory concentration; *f*_u_, unbound fraction; MIC, minimum inhibitory concentration.

Figure 1 summarizes simulations and calculations.

## RESULTS

**FIGURE 2:**
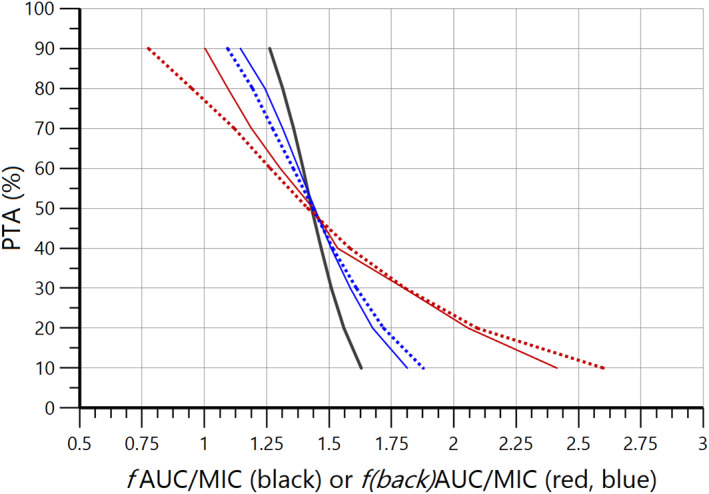
Probability of target attainment (PTA) versus *f*AUC/MIC (units in days) for the free reference distribution and *f*(*back*)AUC/MIC distributions (moderate and high binding scenarios) back computed by scaling the two reference *total*(*AUC/MIC*) distributions derived from the free reference distribution. Black line: PTA of the free reference distribution (no bias). Continuous blue line: PTA of the back calculated *f*(back)AUC/MIC distribution obtained by scaling with *f*_u_ = 0.5 (moderate binding) the reference total(AUC/MIC) distribution generated from the free reference *f*AUC/MIC with an *f*_u_ having an actual log‐normal distribution with mean of 0.5 and a coefficient of variation of 15%. Continuous red line: PTA of the back calculated *f*(back)AUC/MIC distribution obtained by scaling with *f*_u_ = 0.1 (high binding) the reference total(AUC/MIC) distribution generated from the free reference *f*AUC/MIC with an actual uniform distribution of fu ranging from 0.05 to 0.15. Dotted blue line: *f*(back)AUC/MIC as for the continuous blue line but scaling the reference total(AUC/MIC) with a uniform distribution of fu ranging from 0.40 to 0.60 rather than a scalar of 0.5. Dotted red line: *f*(back)AUC/MIC as for the continuous red line but scaling the reference total(AUC/MIC) with an uniform distribution of *f*_u_ ranging from 0.05 to 0.15 rather than a scalar of 0.1. *f*AUC, area under the unbound concentration time curve/minimum inhibitory concentration; *f*_u_, unbound fraction; MIC, minimum inhibitory concentration.

Figure 2 depicts the PTAs obtained for *f*AUC/MIC with the simulated reference distribution, the distribution of the corresponding *total*(*AUC/MIC*) scaled by *f*_u_ of 0.5 or 0.1 and the distribution of *total*(*AUC/MIC*) scaled by an uniform distribution of *f*_u_ (0.4 to 0.6 or 0.05 to 0.15; i.e., of *f*(*back*)AUC/MIC distributions).

Visual inspection of Figure 2 indicates that the magnitude of the bias is greater when the PTA is high (see also the figure generated by R in the annex for PTA greater than 90%). In addition, for a given *f*_u_, the bias is greater when the assumed variability of *f*_u_ is erroneously taken into account for scaling of the back‐computation of *f*AUC/MIC distribution.

Corresponding statistics are given in Table 1.

**TABLE 1:** Selected statistics for the reference free *f*AUC/MIC distribution generated by Monte Carlo Simulations (*n* = 5000) and corresponding values obtained by scaling *total*AUC/MIC with *f*_u_ as a scalar of 0.5 or 0.1 or scaling with uniform distributions of *f*_u_ ranging from 0.40 to 0.60 or from 0.05 to 0.15.

*f*AUC/MIC obtained:	*f*AUC/MIC or *f*(*back*)AUC/MIC (d) for selected PTA% of 90, 50, or 10%			Percentage of subjects achieving *f*(*back*)AUC/MIC of 1.26 (i.e., the reference *f*AUC/MIC for a PTA of 90%)	Dose (mg/kg) required to achieve the reference *f*AUC/MIC (actual PTA%)
	90%	50%	10%		
With reference distribution	1.26	1.43	1.62	90.0	10 (90%)
By scaling *total*AUC/MIC with *f*_u_ = 0.50 (bias%)	1.14 (−9.3%)	1.44	1.81	77.7	11 (89.8%)
By scaling *total*AUC/MIC with *f*_u_ = 0.10 (bias%)	1.00 (−20.5%)	1.44	2.41	63.7	12.5 (89.4%)
By scaling *total*AUC/MIC with *f*_u_ ranging from 0.40 to 0.60 (bias%)	1.09 (−13.4%)	1.44	1.88	71.6	11.5 (89.7%)
By scaling *total*AUC/MIC with *f*_u_ ranging from 0.05 to 0.15 (bias%)	0.78 (−38.5%)	1.41	2.60	60.2	16.5 (90.4%)

*Note*: Units of AUC/MIC are days (d); *f*AUC/MIC was computed with MIC of 1 μg/ml for all simulations. The reference *f*AUC/MIC of 1.26 days (or equivalently 30.2 h) was obtained using Equation 7. The reference *total*AUC/MIC was obtained from the reference distribution using Equation 8, considering an average *f*_u_ of 0.5 with a coefficient of variation of 15% (scenario 1) or an average *f*_u_ of 0.1 with a uniform distribution from 0.05 to 0.15 (scenario 2). This reference *total*AUC/MIC distribution was scaled either by a scalar of 0.5 or 0.1 or by uniform distributions of *f*_u_ (using Equation 9) to yield *f*(back)AUC/MIC distribution. For calculation of the doses to achieve the reference *f*AUC/MIC of 1.26 days (last column), for each increase in the dose of 0.5 mg/kg, the corresponding PTA was determined and the dose retained whose PTA was closest to 90%. In the R‐script, the exact dose was calculated.

Abbreviations: *f*AUC/MIC, area under the unbound concentration time curve/minimum inhibitory concentration; *f*_u_, unbound fraction; PTA, probability of target attainment.

Inspection of Table 1 indicates that the *f*(*back*)AUC/MIC for a PTA of 90% was always negatively biased, yielding lower *f*(*back*)AUC/MIC values than the reference and, therefore, underestimating *f*AUC/MICs. The bias is greater when scaling is conducted with a distribution rather than a scalar. For *f*_u_ of 0.1 and scaling allowing for the variability of *f*_u_, the dose to achieve the reference PTA90% is increased inappropriately by ~60%.

## DISCUSSION

The main conclusion from the present analysis, from both theoretical perspectives and from MCSs, is that the critical values of PTA90%, computed by scaling total plasma concentrations by *f*_u_, expressing the same index in terms of free concentrations, are always biased. This is because total plasma concentrations are ineluctably more variable than their corresponding free concentrations. This, in turn, arises because total plasma concentrations encompass the variability of *f*_u_, the free fraction. In consequence, scaling of total concentrations by the scalar *f*_u_ transfers the whole of this variability to the derived free concentrations.

In the present simulations, for free plasma clearance, a CV of 10% was used. By its nature, free plasma clearance must be less variable than total plasma clearance, because it is not impacted by *f*_u_ variability. The CV% of 10% for clearance, used in the present calculations, was consistent with those reported for antimicrobials which are minimally bound to plasma proteins (i.e., whose variance of total clearance is minimally or not impacted by *f*_u_). For isoniazid,^17^ meropenem,^18^ and amikacin^19^ (for which *f*_u_ > 80%),^20^ the reported BSV for total clearance was less than 20%, and a CV of 10% for free clearance was appropriate for our simulation purposes. Two scenarios, taking into account the degree of binding (*f*_u_ of 0.5 or 0.10), were simulated. It must be recognized that experimental data relating to *f*_u_ distributions are very rare, because a large majority of publications relating to antimicrobial binding to plasma proteins report findings which were conducted on pooled plasma samples and not on plasma from individual patients. When determined in individual patients, the number of subjects (usually healthy volunteers) is often limited (*n* < 15). It is therefore difficult to describe a distribution and it is frequently a mean/median and range of observed values that is reported in the literature. Many MCSs have been performed with *f*_u_ obtained from a marketing authorization monograph^8^ or fixed a priori. For example, an arbitrarily uniform distribution of +/−10% was selected to compute PTA for 13 antimicrobials,^9^ and this, regardless of the *f*_u_ value of each drug. For antimicrobials highly bound to plasma proteins (*f*_u_ < =0.1), we used a uniform distribution, but with a more limited range from 0.05 to 0.15. For antimicrobials moderately bound (*f*_u_ = 50%), a CV of 15% was selected, which is consistent with the variability reported for vancomycin (*f*_u_ = 41.5% with a range of 24–64%)^21^ and for ertapenem with an *f*_u_ of 73.8 +/− 11.6%.^22^

The Q10% of the different distributions (to estimate PTA90%) thereby generated were compared to the Q10% of the reference distributions, generated in the models without binding to plasma proteins. From the results of these simulations, several conclusions follow.

First, Q10% values, obtained from total plasma concentration, are always biased compared to reference values. The bias is minimal for the median/geometric mean (Q50%) but becomes increasingly large when estimating extreme quantiles (Q10% or Q90%) This is due to the slope of the relationship between the value of the index and the corresponding quantiles. These slopes reflect the variance of the back‐calculated distributions, which are always shallower (larger variance) than those of the reference distributions (smaller variance).

Second, given that the biases are linked to the greater variances of the back‐calculated distributions, Q10% and PTA90% will be underestimated. This might lead, during drug development programs, to erroneously proposing higher doses, in order to attain, with the back‐calculated free plasma concentration distributions, the targeted critical values of the PK/PD indices for a PTA of 90%. The greater concern relates to the safety of antimicrobials rather than efficacy.

Third, the bias is greater if the variability associated with *f*_u_ is incorrectly added, instead of removing it during this scaling. For example, selecting a uniform distribution and adding variability in *f*_u_ is strongly discouraged.

Fourth, beta‐lactams are frequently administered by prolonged, constant intravenous infusion, as this enhances efficiency.^16^ It is seldom acknowledged that the equations giving the free AUC and the free Css at equilibrium, following continuous infusion, are algebraically identical but solved with different units (see Equations 2 and 7), the dose (mass units) of Equation 7 being replaced by an infusion rate (mass unit per time) in Equation 2 and the dependent variable now being a Css rather than an *f*AUC distribution. For continuous infusions, the two PK/PD indices (*f*AUC/MIC and %*f*t>MIC) converge operationally, and maintaining a free Css equal to one, two, or five times the MIC over 24 h comprises the same objective, in respect of total dose administered, as obtaining, under equilibrium conditions, an *f*AUC/MIC of 24, 48, or 120 h.

To our knowledge, the issues raised in this paper have not been raised previously and certainly not in respect of the implications we have outlined. The reason for this is that many researchers and clinicians in the field of clinical microbiology continue, mistakenly, to believe that it is the total concentration in vivo, which controls free concentrations, whereas the reverse is true. Indeed, it is counterintuitive to accept that what is true in vitro becomes false in vivo, namely that it is free clearance alone, which controls free concentrations, which in turn controls total plasma concentration.

The considerations and calculations reported in this paper have highlighted the nature and magnitude of the problem and have illustrated its potential clinical importance, when computing PK/PD cutoffs for setting Antimicrobial Susceptibility Testing (AST) clinical breakpoints. However, the issues raised carry much wider implications than for PTA computations. It is ironic to note that very sophisticated population modeling approaches, aimed at optimizing dosages, lead inexorably to conclusions and applications with varying degrees of bias, through confounding free fraction and free concentration. The present data indicate that, for all low extraction ratio drugs—which includes the vast majority of antimicrobials—“*regardless of route of administration*, *and for all drugs administered orally and eliminated primarily by the liver*, *total exposure [of unbound drug] is independent of protein binding and no dosing adjustments will need to be made for real or anticipated changes in fu*”.^23^ Failure to recognize these issues could also be one of the factors underlying the recent controversy over the actual efficiency of therapeutic drug monitoring (TDM) for antimicrobials.^24^, ^25^ Finally, also to be noted as inappropriate, for dosage adjustments, considering covariates derived from the PopPK analysis of total concentrations but that may have no long therapeutic impact when they are directly or indirectly linked solely to plasma protein binding when this binding is linear. The primary goal of antimicrobial therapy is to control exposure of microorganisms to free concentrations of drug and not to optimize total plasma concentrations. This is even more justified when binding to plasma proteins is saturable, as illustrated for ertapenem.^26^

The most appropriate solution to all these problems is simply to measure and model free plasma concentrations (see for example Wilkes et al.^7^ which compares results obtained by modeling free and total plasma concentrations). This can be challenging, as free concentrations can be very low. Furthermore, when measuring low free concentrations, reproducibility may be lower than that of the measured corresponding total concentrations. This potentially introduces analytical variability, which might reduce or even nullify the aim of correcting the bias arising from the variability of *f*_u_. A second option would be to correct the PTAs obtained with total concentrations by taking into account experimental distributions of *f*_u_ (i.e., to deconvolute the mixture of distributions). For this, it is suggested that determining individual *f*_u_ values, and not *f*_u_ from pooled plasma samples, should be encouraged. A third option would be to compute the PTA directly using total and not free plasma concentration, provided that the value of the PK/PD index to be achieved is also expressed in terms of total concentration and determined in a clinical context, as undertaken for vancomycin linezolid and daptomycin.^27^ When these approaches are not possible, acknowledgement should be given of the impact on the accuracy of estimates.

The objective of the present study was not to reach general conclusions on the impact of these more or less biased calculations on the acceptability, or not, of current PTA assessments. It will be for organizations such as CLSI, USCAST, and EUCAST to reassess, or not, their PTA guidelines. It is important to note that, even a bias considered minimal, arising from the variability of *f*_u_ when calculating PTA, can increase the PK/PD cutoff of one dilution, if the decision rule is to accept only PTAs which are strictly equal to or greater than a certain limit (for example = > 90). Indeed, the impact of *f*_u_ variability on the final PK/PD cannot be predicted simply because there is a decision rule that renders no proportionality in the relationship between the size of the bias in *f*_u_ and its impact on the PK/PD cutoff.

A pragmatic solution for those wishing to check whether or not their current PTA assessments might need to be revised would involve conducting a sensitivity study on *total*AUC/MIC to ascertain the influence of the distribution of *f*_u_ on PTA. This can be achieved by making a series of deconvolutions of the *total*AUC/MIC with the distributions of *f*_u_, whose parameters the modeler can select (log‐normal distribution, uniform … and its parameters). Undertaking these deconvolutions is straightforward for some specific *total*AUC and *f*_u_ distributions (namely log‐normal distributions) but requires the writing of nontrivial numerical routines, taking into account the various sources of variability of *total*AUC, namely clearance (i.v. route), Cl/F (extravascular route), and of *f*_u_.

## AUTHOR CONTRIBUTIONS

P.L.T. designed the research, performed the research, and wrote the manuscript. D.C. designed the research and analyzed the data. P.L.T. and P.L. wrote the manuscript. L.P., P.G., A.B.M., and A.F. analyzed the data.

## FUNDING INFORMATION

No funding was received for this work.

## CONFLICT OF INTEREST STATEMENT

The authors declared no competing interests for this work.

## Supporting information


Supporting information S1.
