# Supplementary material for: Biased computation of probability of target attainment for antimicrobial drugs
Source: CPT Pharmacometrics Syst Pharmacol. 2023 Apr 6;12(5):681–9. doi: 10.1002/psp4.12929 (PMC10196429; doi:10.1002/psp4.12929)
Supplement: Supplementary file 1 — Supporting information S1. [file PSP4-12-681-s001.zip › PSP4_12929_PSP-2022-0122-s01.pdf]

```
#####################################################################
# Simulations of the article :
# Biased computation of Probability of Target Attainment for antimicrobial drugs
#####################################################################


n=50000 # MC sample size
#
Dose= 10 # mg/Kg
MIC = 1 # mg/L

# Ln(ClFree)~N(1.9409,0.09975²).
LnClFree=rnorm(n,mean=1.9409,sd=0.09975)
ClFree=exp(LnClFree)
fAUC_MIC=Dose/(ClFree*MIC)


# Scenario 1

#Ln(fu)~N(-0.704272, 0.149166²)
Lnfu=rnorm(n,mean=-0.704272,sd=0.149166)
fu1=exp(Lnfu)
tAUC_MIC1=fAUC_MIC/fu1

# back computation of AUC_free with a fixed fu=0.5
back_AUCfree1=tAUC_MIC1*0.5

# back computation of AUC_free with a random fu uniform on [0.4,0.6]
back_AUCfree1_blurred=tAUC_MIC1*runif(n,min=0.4,max=0.6)



# Scenario 2

# fu~uniform on [0.05,0.15]
fu2=runif(n,min=0.05,max=0.15)
tAUC_MIC2=fAUC_MIC/fu2

# back computation of AUC_free with a fixed fu=0.1
back_AUCfree2=tAUC_MIC2*0.1

#back computation of AUC_free with a random fu uniform on [0.05,0.15]
back_AUCfree2_blurred=tAUC_MIC2*runif(n,min=0.05,max=0.15)



#####################################################################
#                     Plot of PTA (Figure 1)
#####################################################################

x=0.5+(0:2500)/1000 # range of represented fAUC_MIC [0.5,3]
F=ecdf(fAUC_MIC)
PTA_free=100*(1-F(x))
AUC10_free=as.numeric(quantile(fAUC_MIC,probs =0.1))
plot(x,PTA_free,type="l",lwd=2,xlab = c("fAUC/MIC"),ylab=c("PTA(%)"),
     panel.first =grid())
lines(c(0.5,3),c(90,90),col="magenta",lwd=2)

F=ecdf(back_AUCfree1)
PTA_BackFree1=100*(1-F(x))
lines(x,PTA_BackFree1,col="blue",lwd=2)
realPTA_BackFree1=1-F(AUC10_free)
AUC10_BackFree1=as.numeric(quantile(back_AUCfree1,probs =0.1))
```

```
F=ecdf(back_AUCfree1_blurred)
PTA_BackFree1_blurred=100*(1-F(x))
lines(x,PTA_BackFree1_blurred,col="blue",lty=2,lwd=2)
realPTA_BackFree1_blurred=1-F(AUC10_free)
AUC10_BackFree1_blurred=as.numeric(quantile(back_AUCfree1_blurred,probs=0.1))
F=ecdf(back_AUCfree2)
PTA_BackFree2=100*(1-F(x))
lines(x,PTA_BackFree2,col="red",lwd=2)
realPTA_BackFree2=1-F(AUC10_free)
AUC10_BackFree2=as.numeric(quantile(back_AUCfree2,probs =0.1))
F=ecdf(back_AUCfree2_blurred)
PTA_BackFree2_blurred=100*(1-F(x))
lines(x,PTA_BackFree2_blurred,col="red",lty=2,lwd=2)
realPTA_BackFree2_blurred=1-F(AUC10_free)
AUC10_BackFree2_blurred=as.numeric(quantile(back_AUCfree2_blurred,probs=0.1))


###################################################################
# Column 1 of table 1 :fAUC/MIC (d) for selected PTA%
###################################################################

# Actual values of tAUC/MIC for selected PTA%
  -quantile(-fAUC_MIC,probs = c(0.1,0.5,0.9))

# By Scaling tAUC/MIC with fu=0.5
  -quantile(-back_AUCfree1,probs = c(0.1,0.5,0.9))

# By Scaling tAUC/MIC with fu uniform on [0.4,0.6]
  -quantile(-back_AUCfree1_blurred,probs = c(0.1,0.5,0.9))

# By Scaling tAUC/MIC with fu=0.1
  -quantile(-back_AUCfree2,probs = c(0.1,0.5,0.9))

# By Scaling tAUC/MIC with fu uniform on [0.05,0.15]
  -quantile(-back_AUCfree2_blurred,probs = c(0.1,0.5,0.9))

######################   Bias

# bias (%) when tAUC/MIC is scaled with fu=0.5
  100*(AUC10_BackFree1-AUC10_free)/AUC10_free

# bias (%) when tAUC/MIC is scaled with fu uniform on [0.4,0.6]
  100*(AUC10_BackFree1_blurred-AUC10_free)/AUC10_free

# bias (%) when tAUC/MIC is scaled with fu=0.1
  100*(AUC10_BackFree2-AUC10_free)/AUC10_free

# bias (%) when tAUC/MIC is scaled with fu uniform on [0.05,0.15]
  100*(AUC10_BackFree2_blurred-AUC10_free)/AUC10_free


###################################################################
# Column 2 of table 1 :fAUC/MIC (d) for selected PTA%
# the first PTA is by definition equal to 90%
###################################################################

# By Scaling tAUC/MIC with fu=0.5
  realPTA_BackFree1*100

# By Scaling tAUC/MIC with fu uniform on [0.4,0.6]
  realPTA_BackFree1_blurred*100

# By Scaling tAUC/MIC with fu=0.1
```

```
  realPTA_BackFree2*100

# By Scaling tAUC/MIC with fu uniform on [0.05,0.15]
  realPTA_BackFree2_blurred*100


###################################################################
# Column 3 of table 1 :Dose (mg/kg) required to get a PTA of 90% to achieved
# the reference fAUC/MIC
###################################################################

# By Scaling tAUC/MIC with fu=0.5
  Dose*AUC10_free/AUC10_BackFree1

# By Scaling tAUC/MIC with fu uniform on [0.4,0.6]
  Dose*AUC10_free/AUC10_BackFree1_blurred

# By Scaling tAUC/MIC with fu=0.1
  Dose*AUC10_free/AUC10_BackFree2

# By Scaling tAUC/MIC with fu uniform on [0.05,0.15]
  Dose*AUC10_free/AUC10_BackFree2_blurred
```
